# [WITHDRAWN] Overlapping Targets of Shh and Gata3 during Craniofacial Development

**DOI:** 10.21203/rs.3.rs-2973064/v2

**Published:** 2023-11-06

## Abstract

The full text of this preprint has been withdrawn by the authors while they make corrections to the work. Therefore, the authors do not wish this work to be cited as a reference. Questions should be directed to the corresponding author.

